# Patterns of serial rib fractures after blunt chest trauma: An analysis of 380 cases

**DOI:** 10.1371/journal.pone.0224105

**Published:** 2019-12-19

**Authors:** Christian Liebsch, Tina Seiffert, Markus Vlcek, Meinrad Beer, Markus Huber-Lang, Hans-Joachim Wilke

**Affiliations:** 1 Institute of Orthopaedic Research and Biomechanics, Trauma Research Centre Ulm, Ulm University, Ulm, Germany; 2 Department of Diagnostic and Interventional Radiology, Ulm University Medical Centre, Ulm, Germany; 3 Institute for Clinical and Experimental Trauma Immunology, Ulm University Medical Centre, Ulm, Germany; Duke University School of Medicine, UNITED STATES

## Abstract

Rib fractures represent the most common bone fracture, occurring in 10–20% of all blunt trauma patients and leading to concomitant injuries of the inner organs in severe cases. The purpose of this study was to identify specific serial rib fracture patterns after blunt chest trauma. 380 serial rib fracture cases were investigated. Fractures were assigned to five different locations within the transverse plane. Rib level, fracture type, and dislocation grades were recorded and related to the cause of accident. In total, 3735 rib fractures were identified (9.8 per patient). 54% of the rib fractures were detected on the left thorax. Rib fracture distribution exhibited a hotspot at rib levels 4 to 7 in the lateral and posterolateral segments. On average, most rib fractures occurred in crush/burying injuries (15.8, n = 13) and pedestrian accidents (12.8, n = 13), least in car/truck accidents (8.9, n = 75). In the car/truck accident group, 47% of all rib fractures were in the lateral segment, in case of frontal collision (n = 24) even 60%. Fall injuries (n = 141) entailed mostly posterolateral rib fractures (35%). In case of falls >3 m (n = 45), 48% more rib fractures were detected on the left thorax. In cardiopulmonary resuscitation related serial rib fractures (n = 33), 70% of all rib fractures were located anterolaterally. Infractions were the most observed fracture type (44%), followed by oblique (25%) and transverse (18%) fractures, while 46% of all rib fractures were dislocated (15% ≥ rib width). Serial rib fractures showed distinct fracture patterns depending on the cause of accident. When developing a serial rib fracture classification system, data regarding patterns, fracture types, dislocation grades, and associated fractures should be included.

## Introduction

Rib fractures are common injuries after blunt chest trauma, occurring in 60–80% of all cases [1,2]. In general, rib fractures represent the most frequent type of bone fractures, being observed in about 10–20% of all trauma patients [3–5]. More than 20% of these patients suffer from chronic pain in the thoracic region, while rib fractures are mostly treated conservatively, thus often leading to painful pseudarthrosis [6–8].

In about 50% of all rib fracture cases, three or more consecutive ribs are broken, also referred to as serial rib fractures [1,9]. Combinations of multiple rib fractures can result in a distinct loss of integrity of the rib cage as well as in a severe instability of the chest wall, finally leading to thoracic deformities and movement pain in the chest [5,8,10]. In most cases of thoracic trauma, rib fractures do not arise isolatedly, but entail concomitant injuries [11]. In severe cases, the rib fragments harm the inner organs, such as the lungs, the liver, the kidneys, or the spleen [1,3,9,11,12]. Since rib fractures are often related to specific intrathoracic and intraabdominal traumas, predictions of the probability, progression, and complication rate of the inner organ injuries are possible [12–15]. Detailed knowledge about the fracture patterns of serial rib fractures is therefore essential to improve the treatment strategy of these types of injury.

Despite the high incidence of rib fractures, the relatively high morbidity, and therefore the need for consistent proceedings in the clinical treatment of rib fractures, there is still no established classification system for fractures of the rib cage. Without a standardized classification system there will most probably be a lack of consistency regarding the documentation of rib fractures, leading to an insufficient comparability of clinical and scientific findings and thus to inadequate treatments. Existing classification systems for long bones include fracture mechanisms, fracture types, and dislocation grades. Previous studies on rib fractures, however, mainly focused on specific groups of patients or causes of accidents when investigating incidences or fracture localizations, for instance in the context of cardiopulmonary resuscitations [16–19] or traffic accidents [20–24]. In most of these studies, the specific fracture patterns, including fracture types or dislocation grades, were not investigated. In the study of Crandall et al. [22], rib cages of cadaver specimens were radiologically analyzed after simulating car crashes to evaluate the effects of airbags and seatbelts on the observed fracture patterns. This was redone by Lee et al. [24] using a radiological database of car occupants and a method to determine the fracture location from CT images, including five different fracture locations per rib, which was developed by Ritchie et al. [25]. To the authors’ best knowledge, however, there is still no larger scale study, which investigated the fracture patterns, fracture types, dislocation grades, and associated fractures of the various cases of blunt chest trauma in daily clinical practice using CT scans.

Therefore, the main purposes of this study were to identify (1) specific serial rib fracture patterns, (2) possible correlations between causes of accidents and fracture patterns, (3) specific rib fracture types and dislocation grades after blunt chest trauma, and (4) possible rib fracture patterns in combination with associated vertebral, sternal, or clavicle fractures using a large heterogeneous patient collective.

## Materials and methods

### Data acquisition

All cases of serial rib fractures in the period from August 2008 to December 2015 were extracted from the radiological database of our university hospital using keyword search. Serial rib fractures had been defined previously as three or more consecutive rib fractures by the radiology department of our university hospital according to the ICD-10 code 22.4. All scans were performed in a standardized manner using the same CT device (Philips Brilliance iCT 256, Philips Healthcare, Cleveland, USA). Inclusion criteria for our study were the presence of (1) at least one serial rib fracture per patient, (2) a complete CT dataset of every serial rib fracture patient, and (3) a complete patient dataset including age, gender, and cause of accident. Patients aged less than 18 years were not included in the study. Finally, 380 patients were included in this study following these criteria. Using Microsoft Excel (Excel 14.7.0, Microsoft Corp., Redmond, USA), the patient data, the number of rib fractures, the affected rib levels, as well as same-level associated fractures of the sternum, thoracic spine, and clavicle were recorded for each patient. Isolated rib fractures, which were not part of the serial rib fractures, were also taken into account during data acquisition, as well as multiple fractures of multiple ribs, also referred to as flail chest. Based on the available anamnestic data in the clinical reports, the single cases were assigned to eleven groups with respect to the specific cause of accident (Table 1). Fracture positions, fracture types, and dislocation grades, which represent common parameters in classification systems of long bone fractures, were determined from the transverse plane CT scans for each patient.

**Table 1:** Subgroups of the patients according to the cause of accident.

Group no.	Cause of accident	n	Fpp
1	Cardiopulmonary resuscitation	33	9.1
2	Car/truck accident	75	8.9
3	Motorcycle accident (collisions and falls)	42	9.9
4	Bicycle accident (collisions with vehicle or stationary object)	14	10.7
5	Pedestrian accident (collisions with vehicle)	13	12.8
6	Fall from bicycle	21	9.0
7	Fall from low height (< 3 m)	96	9.7
8	Fall from great height (≥ 3 m)	45	10.8
9	Squashing/burying accident	13	15.8
10	Others (iatrogenic fractures, osteoporosis-related fractures, etc.)	16	7.9
11	Unclear information	12	8.3
	Overall collective	380	9.8

n = group size, fpp = fractures per patient.

### Fracture localization method

**Fig 1:**
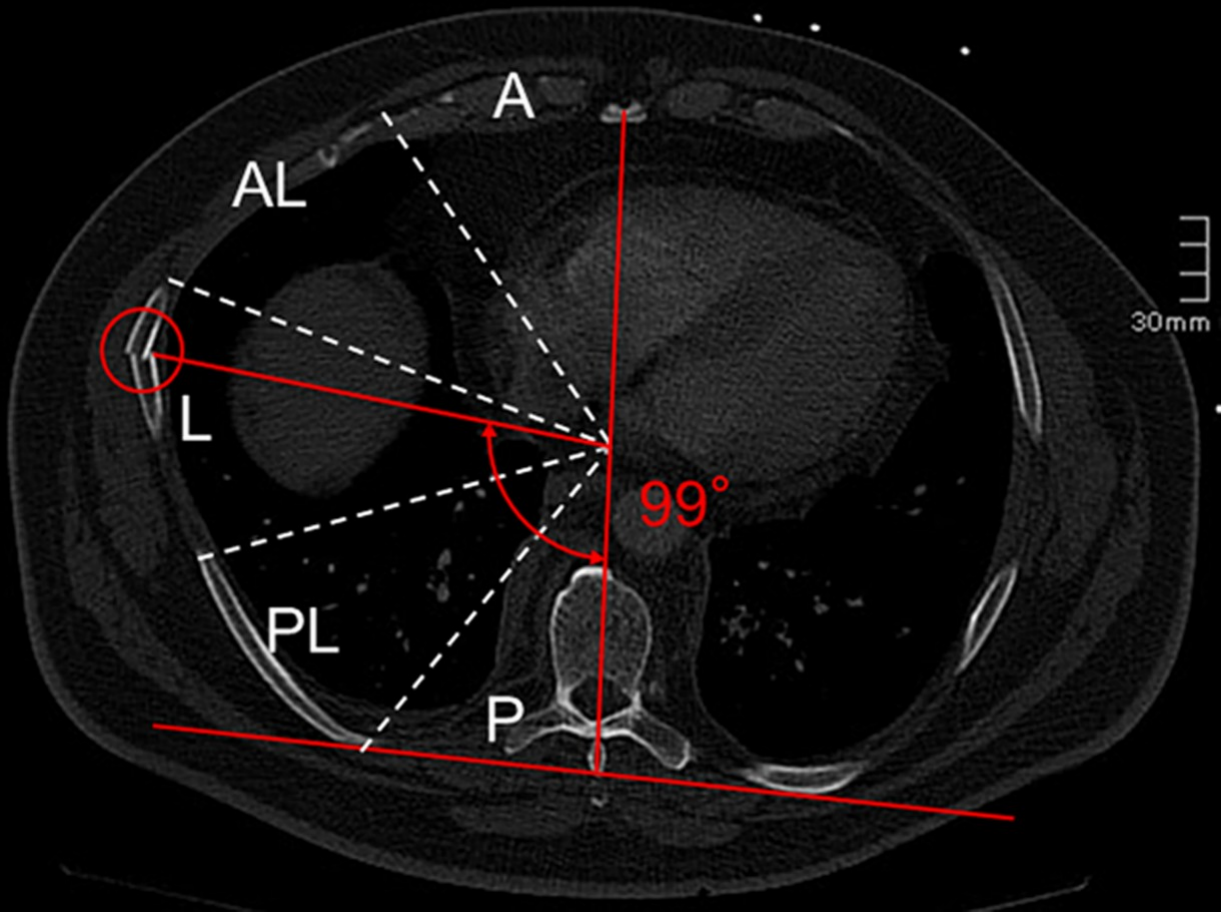
Exemplary illustration of the rib fracture localization using the method of Ritchie et al. (2006).

The evaluation of the fracture positions was performed using an established method by Ritchie et al. [25], calculating the angular position of the fracture gap from transverse CT slices using the software ImageJ (ImageJ 1.49, National Institutes of Health, Maryland, USA). Using this method, the fracture positions were assigned to one of five sections within the transverse plane, each having an angle of 36° (Fig 1). In case of the first rib, the fractures were generally assigned to three different sections due to the small rib size, according to the method of Ritchie et al. Before data evaluation, the intra-rater reliability of this method was tested for the person who evaluated the data afterwards using ten different samples and ten ratings per sample, showing an almost perfect agreement of the measurements (Fleiss’ κ = 0.97, mean deviation of 1.6° in the Bland-Altman plot). Using the evaluated data, heat maps were generated based on the method of Lee et al. [24]. Additionally, hotspots within the heat maps were defined as the smallest areas which included at least 50% of all fractures within the respective side of the rib cage.

### Ethics

Our study was approved by the independent local ethics committee of the University of Ulm (Internal no. 55/16). All patient data was anonymized at the beginning of data acquisition by assigning sequential numbers to the single cases. Informed consent of the patients was therefore not required according to the responsible ethics committee.

## Results

In total, the 380 patients exhibited 3735 rib fractures (9.8 rib fractures per patient). The mean age of the patients was 60 years, ranging from 18 to 94 years. 287 patients were male (76%), 93 (24%) were female. 119 patients had associated fractures of the thoracic spine, sternum, or clavicle (31%). The number of fractures per patient ranged from three (n = 24) to 33 (n = 1), gradually decreasing from four fractures per patient (n = 38) to 20 fractures per patient (n = 3), while there was no distinct effect of age or sex on the fractures per patient ratio.

**Fig 2:**
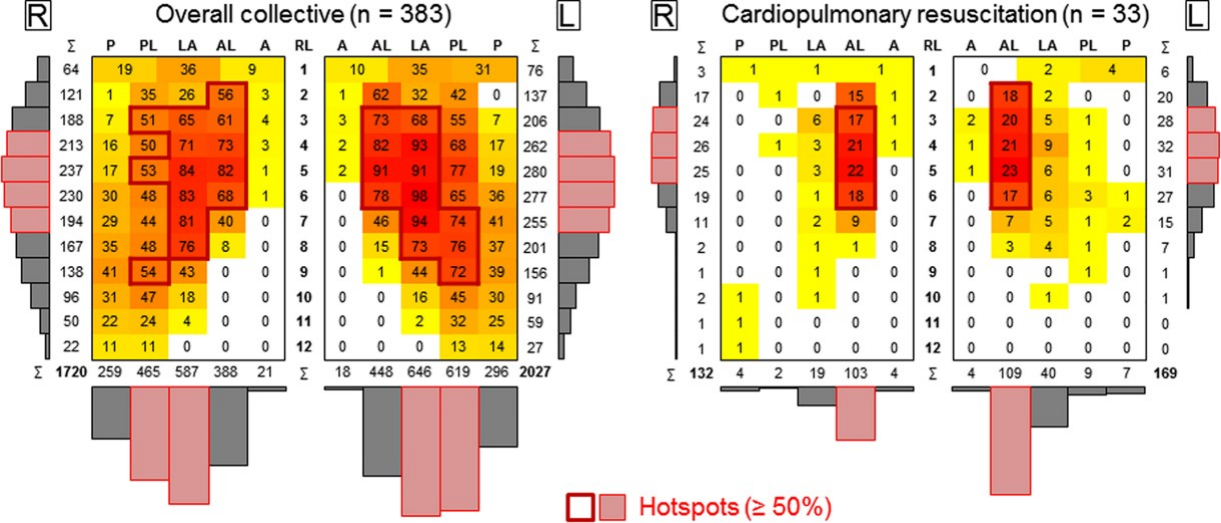
Heat maps illustrating the rib fracture distributions on the rib cage in frontal view for the overall collective and the cardiopulmonary resuscitation group. R/L = right/left side of the rib cage, RL = rib level, P = posterior, PL = posterolateral, LA = lateral, AL = anterolateral, A = anterior.

In the overall collective, the rib fracture number increased from rib level 1 to rib level 5 and decreased from level 5 to rib level 12, generally showing a bell-shaped distribution of the fractures between the rib levels 1 and 10 on both sides of the rib cage (Fig 2). 54% of all fractures were found on the left side, 46% on the right side. Most rib fractures were detected in the lateral section on both sides of the rib cage, followed by the posterolateral and anterolateral sections. More than 50% of all rib fractures were between the rib levels 4 and 7 and in the lateral and posterolateral sections, respectively, leading to oblique running hotspot areas from anterolateral-cranial to posterolateral-caudal on both sides of the rib cage.

In the cardiopulmonary resuscitation (CPR) group, the most distinct fracture pattern of all subgroups was found (Fig 2). 70% of the rib fractures were detected in the anterolateral section and more than 50% between the rib levels 3 and 5, resulting in the smallest hotspot area of all examined groups within the present study. 56% of the rib fractures were detected on the left side of the rib cage and 44% on the right side, while the proportion of anterolateral rib fractures was higher on the right side of the rib cage (78%) than on the left side (64%).

**Fig 3:**
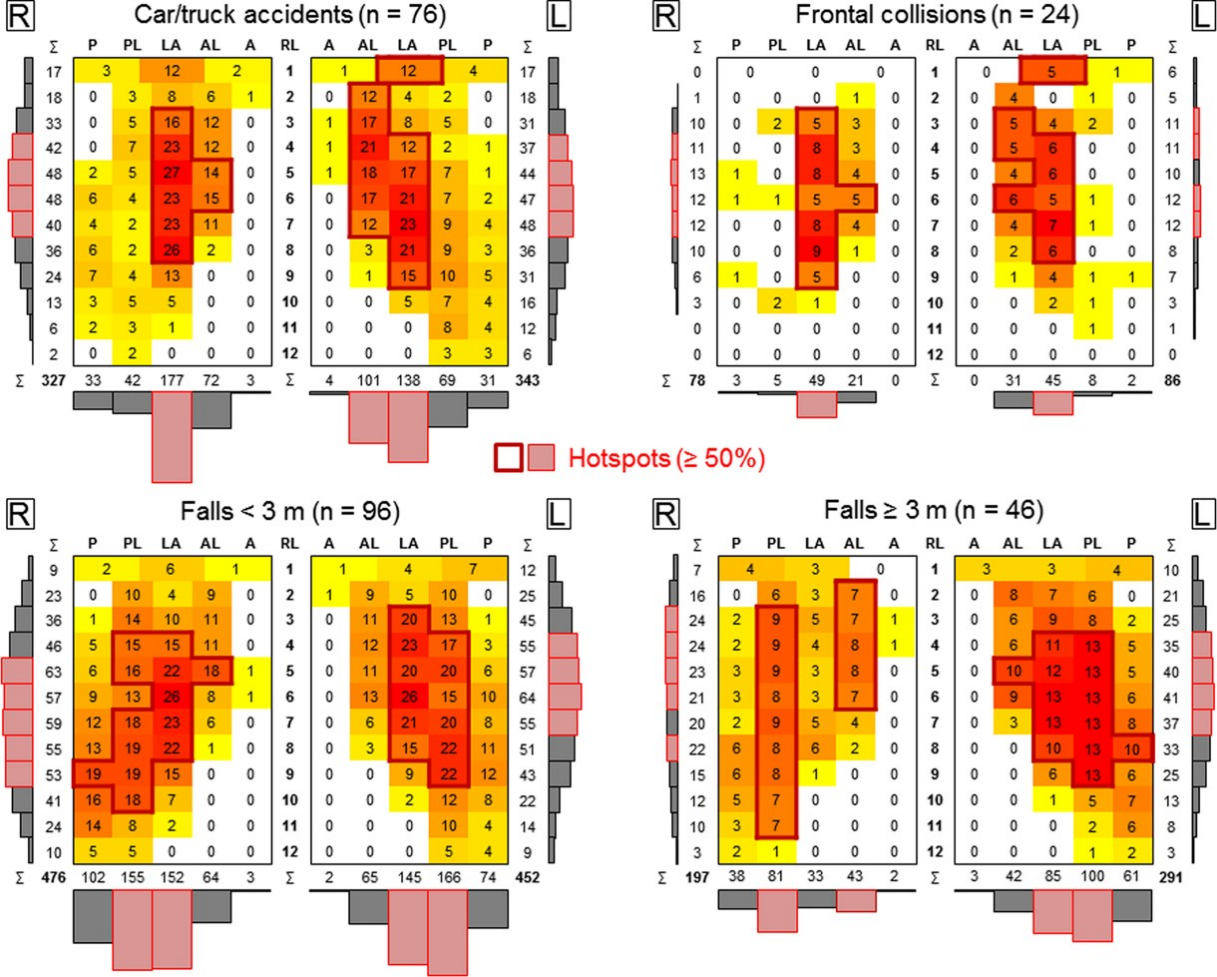
Heat maps illustrating the rib fracture distributions on the rib cage in frontal view for the car/truck accident group and its subgroup of frontal collisions as well as the fall groups including falls from low height and falls from great height. R/L = right/left side of the rib cage, RL = rib level, P = posterior, PL = posterolateral, L = lateral, AL = anterolateral, A = anterolateral.

In the car/truck accident group, more than 50% of the rib fractures were between the rib levels 4 and 7 on both sides of the rib cage (Fig 3). Furthermore, side differences were detected in this group regarding the fracture position. On the right side of the rib cage, more than 50% of the rib fractures were in the lateral section, while on the left side, lateral rib fractures accounted for 40%, followed by anterolateral fractures with 29%. Looking at the subgroup of solely frontal collisions, the fracture pattern became more distinct with more than 50% of all rib fractures being in the lateral section on both sides of the rib cage, but still exhibiting a higher proportion of anterolateral rib fractures on the left side of the rib cage (36% vs. 27%, Fig 3).

The group of falls from low height (< 3 m) exhibited mostly posterolateral and lateral fractures on both sides of the rib cage (35% posterolateral, 32% lateral, Fig 3). Regarding the affected rib levels, side differences were detected, since the rib fractures on the right side of the rib cage were located more caudally (> 50% between rib levels 5 and 9) compared with the left side (> 50% between rib levels 4 and 7), generally showing a more indistinct fracture pattern. In the group of falls from great height (≥ 3 m), the fracture pattern on the right side of the rib cage exhibited two disconnected hotspots with more than 50% of all rib fractures in the posterolateral (41%) and anterolateral (22%) sections and a more uniform distribution along the rib levels 2 to 11 (Fig 3). The fracture pattern on the left side of the rib cage was more compact and centralized with more than 50% of the rib fractures in the posterolateral (34%) and lateral (29%) sections as well as more than 50% between the rib levels 4 and 7. In general, more rib fractures were located on the left side of the rib cage (60%) than on the right side (40%), whereas in the group of falls from low height, the number of fractures was almost the same comparing the left (51%) and right (49%) side of the rib cage, respectively.

In the remaining groups, which are summarized in the supplementary file within the electronic version of this article, more heterogeneous fracture distributions were found, especially in the squashing/burying accident group. In this group, the highest fractures per patient (fpp) ratio was found (15.8 fpp), followed by the pedestrian accident group (12.8 fpp), the group of falls from great height (10.8 fpp) and the bicycle accident group (10.7 fpp). The lowest fpp ratios were detected in the car/truck accident group (8.9 fpp), the fall from bike group (9.0 fpp), and the CPR group (9.1 fpp). In the motorcycle accident group, the bicycle accident group, and the pedestrian accident group, the rib fracture number was higher on the left side of the rib cage (~ 60%) compared with the right side (~ 40%), while in both the falls from bicycle and squashing/burying accident groups, the number was almost even comparing both sides of the rib cage.

**Fig 4:**
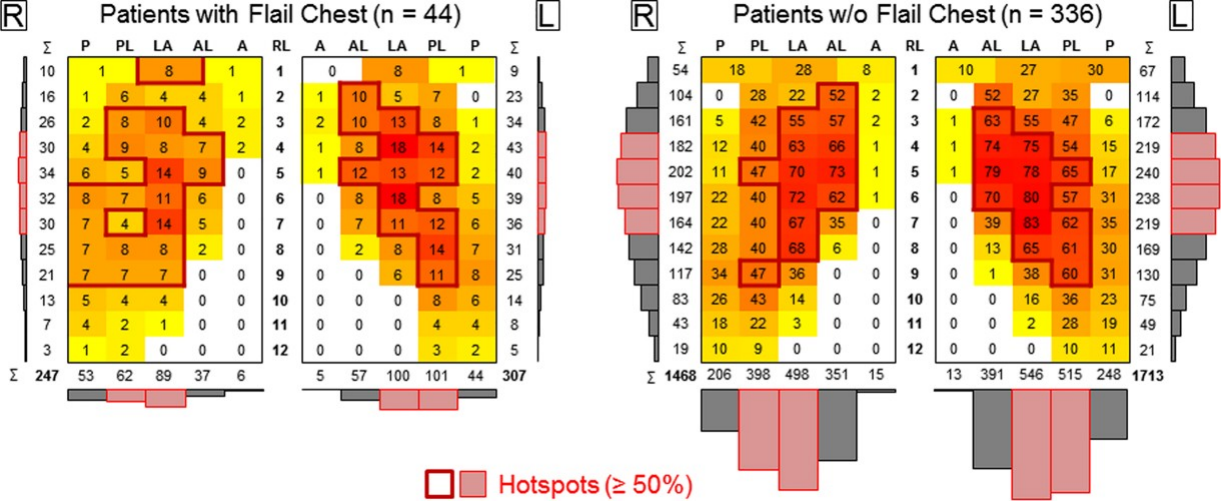
Heat maps illustrating the fracture distribution on the rib cage in frontal view for patients with and without flail chest. R/L = right/left side of the rib cage, RL = rib level, P = posterior, PL = posterolateral, L = lateral, AL = anterolateral, A = anterolateral.

Patients exhibiting flail chest (n = 44) showed overall a similar fracture distribution compared to patients without flail chest (n = 336), considering the lower amount of cases and thus higher inhomogeneity in the fracture pattern (Fig 4), while fracture per patient ratio was distinctly higher (12.6 fpp) than in the group of patients without flail chest (9.5 fpp).

Flail chests were especially detected in the squashing/burying accident group (23%), where also fractures per patient rate and thorax drainage rate were highest, followed by the bicycle accident group (20%), and the group of falls from low height (19%) (Table 2). Flail chest, however, did not explicitly correlate with fractures per patient ratio and clinical parameters such as the average period of hospital or intensive care unit stay, thorax drainage rate, or average duration of assisted respiration. Average hospital and intensive care unit stay as well as average duration of assisted respiration were highest in the pedestrian group, which also exhibited the second highest thorax drainage rate, while at the same time showing below-average flail chest rate of 7% (Table 2).

**Table 2:** Clinical parameters and patient outcomes regarding the cause of accident.

Group	Flail chest (%)	Fpp	Avg. hospital stay (d)	Avg. ICU stay (d)	Thorax drainage (%)	Avg. duration of assisted respiration (d)
Cardiopulmonary resuscitation	6	9.1	21	8	12	9
Car/truck accident	6	8.9	25	13	51	10
Motorcycle accident	10	9.9	23	16	54	13
Bicycle accident	20	10.7	21	9	40	15
Pedestrian	7	12.8	37	19	67	33
Fall from bicycle	10	9.0	15	11	52	11
Fall from low height (< 3 m)	19	9.7	16	9	33	6
Fall from great height (≥ 3 m)	11	10.8	21	10	33	6
Squashing/burying accident	23	15.8	32	12	77	9
Others	6	7.9	13	7	39	6
Unclear information	0	8.3	1	1	0	1
Overall collective	11	9.8	21	12	40	10

Fpp = fractures per patient, ICU = Intensive care unit.

Four main types of fracture were identified in the overall collective: Transverse fractures, oblique fractures, multifragment fractures, and infractions (Table 3). Infractions were generally the most observed fracture type (44%), followed by oblique fractures (25%), and transverse fractures (18%). Multifragment fractures accounted for 10% of all rib fractures. 4% of the evaluated rib fractures exhibited inconclusive fracture types, which were interpreted as callus formation. 32% of all infractions were found in the anterolateral section, where their proportion accounted for 64%. About 70% of all oblique and transverse rib fractures were detected in the combined lateral and posterolateral sections, where their ratios were about 30% and 20%, respectively. 67% of all multifragment fractures were found in the combined lateral and posterolateral sections, while their proportions were highest in the posterolateral and posterior sections with 12% and 15%, respectively. A distinct effect of the cause of accident on the fracture type was not detected.

**Table 3:** Fracture types, dislocation grades, as well as same-level and same-side associated rib fractures in the overall collective of the present study.

Category	Classification	Total number	Percentage
Fracture types	Transverse fractures	662	18%
	Oblique fractures	940	25%
	Multifragment fractures	356	10%
	Infractions	1632	44%
	Callus formation	145	4%
Dislocation grades	No dislocation	2015	54%
	Dislocations < rib width	1174	31%
	Dislocations ≥ rib width	546	15%
Same-level and same side associated rib fractures	RFAW vertebral body fractures	170	5%
	RFAW transverse process fractures	103	3%
	RFAW sternal fractures	469	13%
	RFAW clavicle fractures	8	0%
Overall collective	All evaluated rib fractures	3735	100%

RFAW = Rib fractures associated with.

Three grades were chosen to classify the dislocation of the rib fractures: No dislocation, dislocations smaller than the respective rib width, and dislocations greater than the respective rib width (Table 3). 54% of all rib fractures were not dislocated. About 60% of these fractures were found in the anterolateral and lateral sections on both sides of the rib cage. Rib fractures with dislocations smaller than the rib width (31%) and rib fractures with dislocations greater than the rib width (15%) were mostly located in the lateral and posterolateral sections (~ 70% and ~ 75%, respectively).

72 patients (19%) had associated fractures of the thoracic vertebrae, while 54 patients (14%) exhibited fractures of the vertebral body and 25 patients (7%) fractures of the transverse process. 50 patients (13%) had sternal fractures and 14 patients (4%) showed fractures of the clavicle. In the bicycle accident group, the highest proportion of patients with associated fractures was detected (57%), followed by the car/truck accident group (46%), and the motorcycle accident group (41%). Of the 54 patients with associated vertebral body fractures, 38 patients had associated vertebral body fractures at the same level and the same side as the respective rib fracture, overall showing 170 same-level and same-side associated rib fractures (Table 3). 35% of these rib fractures were located in the posterolateral section, 27% in the lateral section, and 24% in the posterior section. Of the 25 patients with fractures of the transverse process, 19 patients showed overall 103 same-level and same-side associated rib fractures. 34% of these rib fractures were located in the posterolateral section and 32% in the posterior section. All of the 50 patients with fractures of the sternum exhibited overall 469 same-level associated rib fractures. 37% of these rib fractures were located in the anterolateral section and 36% in the lateral section. Of the 14 patients with clavicle fractures, 6 patients showed overall 8 same-level and same-side associated rib fractures. 6 of these 8 rib fractures (75%) were located in the lateral section of the ribs.

All heat maps which were analyzed in the present study are retrievable from the supplementary material file S1 Dataset.

## Discussion

**Fig 5:**
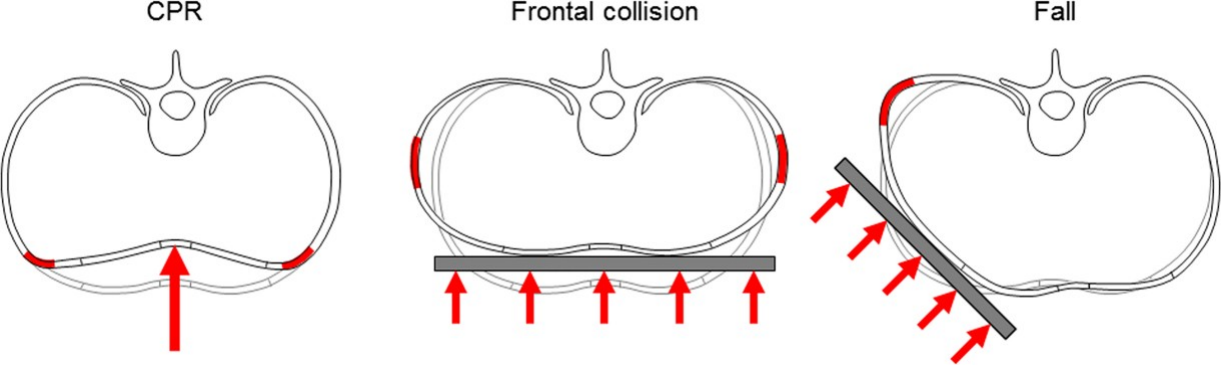
Possible fracture mechanisms causing the detected specific fracture patterns in cases of cardiopulmonary resuscitations (~70% anterolateral rib fractures), frontal collisions (~60% lateral rib fractures), and falls (~40% posterolateral rib fractures).

The findings of the present study indicate that serial rib fractures generally show specific fracture patterns. The overall collective exhibited diagonally running fracture hotspots on both sides of the rib cage, each in the middle third and from cranial/anterolateral to caudal/posterolateral, which therefore can be seen as the most affected areas of the rib cage regarding rib fractures. Furthermore, the rib fracture patterns seem to depend on the cause of accident and thus on specific fracture mechanisms. The high amount of anterolateral rib fractures in case of CPRs could be explained by the concentrated loads on the sternum which leads to high stress peaks in the anterior parts of the ribs due to the strong deformation of the costal cartilage. During frontal car collisions, however, the ribs presumably undergo high bending deformations in the lateral part of the ribs due to higher impact surfaces, which might explain the high proportion of lateral fractures in this subgroup. The high amounts of posterolateral and lateral fractures in case of falls indicate that the loading happened from an oblique angle in many cases and led to high bending stress in the posterolateral section where usually the strongest curvature of the rib exists (Fig 5).

The affected rib level, on the other hand, did not appear to be distinctly influenced by the different causes of accident, with the exception of CPR related rib fractures, which were located more cranially, and fall related rib fractures, which were located more caudally on the right side of the rib cage. In the overall collective and in most subgroups, however, the rib levels 4 to 7 exhibited more than half of all rib fractures, indicating that rib fracture distribution represents a function of rib length, since the rib levels 5 to 7 are the longest ribs and the single rib lengths show a similar distribution compared to the fracture distribution in the present study [26]. Because of their length, these ribs are probably more exposed and thus at higher risk for being mechanically loaded and finally fractured.

The specific fracture patterns of the single groups can be mainly explained by the boundary conditions relating to the causes of accidents. Patients after CPR showed the most distinct fracture pattern, which is probably caused by the well-definable boundary conditions during the procedure of resuscitation, explaining similar fracture patterns and fracture per patient ratios in previous studies [16,18,27–30]. The few rib fractures, which were not in the area of the fracture hotspot of this group, most probably resulted from injuries before resuscitation and could not be distinctly separated in some cases. Patients with rib fractures after car or truck accidents had rib fractures on both sides of the rib cage, while the fractures were mostly in the lateral section on the right side and in both the anterolateral and lateral sections on the left side of the rib cage, indicating that the trauma mechanisms were affected by non-frontal loadings in some cases, which was already shown in previous studies [22,24]. These mechanisms could include the effects of seatbelts or lateral impacts, which were not investigated in further subgroups in the present study, since the documentation was in some cases not detailed enough for the car/truck accident mechanism and the case numbers of these subgroups would have been generally too low to create conclusive results. Therefore, side differences due to inversely worn seatbelts of drivers and passengers were not evaluated. The fracture patterns of the other traffic injury groups, i.e. motorcycle, bicycle, and pedestrian accidents, generally showed more heterogeneous fracture patterns, indicating higher variations in the fracture mechanisms within these groups, including collisions and falls or combined traumas, which were also not precisely deducible from the documentation. Since there were more rib fractures on the left side of the rib cage in each of these groups, however, an effect of the impact direction due to road traffic regulations can be expected. The rib fracture patterns resulting from falls, which showed slight differences regarding the causes of accident, could also be affected by the specific boundary conditions at the moment of trauma. Falls from bicycle exhibited a hotspot in the posterolateral section of the left rib cage side, while there was no side difference in the group of falls from low height and a hotspot in the posterolateral section of the right rib cage side in the group of falls from great height, indicating possible effects of the patients’ body movement and posture during the accident sequence. Additionally, increasing height could lead to higher impact and inertial loads. The specific fracture pattern after falls, generally showing straight-line fracture arrangements from cranial to caudal, was in accordance with previous studies [31–33]. Finally, the rib fracture pattern of squashing or burying accidents, which was the most heterogeneous in the present study, could be explained by the complex loading mechanisms, which most probably consisted of compression loads from different directions.

In the present study, infractions were the most detected fracture type, which probably result from low force impacts. Multifragment fractures, on the other hand, are most likely caused by high impact loads, while transverse and oblique fractures potentially are related to the impact direction and the angular position of the ribs after medium impact loadings. In fact, infractions of the ribs were mainly found in the anterolateral sections of the rib cage, for instance in case of CPRs, which are not caused by high loads, while multifragment rib fractures were mainly located in the posterolateral sections, indicating high impact loads, for instance in the case of falls from great height. Transverse rib fractures were generally located more cranially than oblique rib fractures, which could be related to the more horizontal position of the upper ribs and the more diagonal position of the lower ribs. Moreover, transverse rib fractures were more anteriorly than oblique rib fractures, which could be explained by the smaller curvature of the ribs in the anterior rib sections. Furthermore, the fracture type could be influenced by the specific morphology or the material properties of the single ribs, since the fracture types of different ribs varied for the same loading conditions in experimental testings [34].

Approximately half of all rib fractures were dislocated, especially in combination with oblique and multifragment fractures and in the posterolateral section, where the highest proportion of dislocations greater than rib width was found. In contrast, the highest proportion of non-dislocated rib fractures was detected in the anterolateral section, for instance after CPR as in a previous study [28], indicating a high positive correlation between impact loading and dislocation grade. Moreover, rib fractures which were combined with fractures of the thoracic vertebrae, the sternum, or the clavicle were mainly found in sections close to these associated fractures. Therefore, the impact loading is probably transferred to the adjacent structures in many cases and does not solely damage an isolated structure. Fractures of the transverse processes, for instance, potentially are caused by leverage due to the antero-posterior compression forces of the ribs in frontal impacts.

The average number of rib fractures per patient within the present study (9.7) was considerably higher than in previous studies investigating epidemiological aspects of rib fractures, where the average number was between two and six rib fractures per patient [12,24,35]. This discrepancy could be explained by the inclusion criteria of the present study, since solely serial rib fracture cases, which exhibited at least three rib fractures, were analyzed, neglecting cases of single rib fractures. An effect of the age on the fractures per patient ratio could neither be found in the present study nor in previous studies. The average age of the patients (59 years), however, was slightly higher in the present study compared to previous studies [3,9,36]. The higher proportion of male patients within the present study (76% of all patients, 77% of all rib fractures) was also shown in previous studies [3,9,35–37]. Furthermore, the relative group sizes regarding the causes of accident were similar in previous studies, with traffic accidents and falls generally representing the largest groups [1,3,9,38].

The present study entails some limitations, which especially result from its retrospective study design. The loading mechanisms of the single accidents were in many cases not clearly determinable, since there were probably various boundary conditions which were not precisely documented in the clinical reports or which could not be reconstructed after the accident. In case of traffic accidents, for instance, collisions from multiple directions, falls on different ground surfaces, and varying involved safety devices were combined within specific groups according to their vehicle type, making interpretations of the results difficult in these subgroups. Moreover, the data could be affected by selection bias, since combined traumas could not be completely excluded, for instance in CPR related injuries. Therefore, statistical evaluations of the collected data were not performed in the present study. Moreover, clinical data could not be correlated with the fracture patterns of the single subgroups because of highly heterogeneous and possibly biased clinical data, since it could not be assured that the rib fractures were causing these parameters. The same applies for associated organ injuries of liver, spleen, heart, etc., which could not be clearly assigned to fractures of the ribs as initiators in a large number of cases. These correlations should be investigated in future studies using a prospective study design.

## Conclusions

In conclusion, the results of the present study show that there are specific rib fracture patterns after blunt chest trauma. Our results should be confirmed by prospective and experimental studies to reduce the effects of inconclusive boundary conditions. When developing a serial rib fracture classification system, causes of accident, fracture types, dislocation grades, and associated sternal, spinal, and clavicle fractures should be included as diagnostic parameters for the complete description of serial rib fractures.

## Supporting information

S1 Dataset Supplementary material. Heat maps of all investigated subgroups.
(PDF)
