# Supplementary material for: Patterns of serial rib fractures after blunt chest trauma: An analysis of 380 cases
Source: PLoS One. 2019 Dec 19;14(12):e0224105. doi: 10.1371/journal.pone.0224105 (PMC6922429; doi:10.1371/journal.pone.0224105)
Supplement: S1 Dataset — Heat maps of all investigated subgroups. (PDF) [file pone.0224105.s001.pdf]

Supplementary material

List of abbreviations:

- n = Number of patients
- fpp = Fractures per patient ratio

- R = Right side of the rib cage
- L = Left side of the rib cage

- RL = Rib level

- P = Posterior section
- PL = Posterolateral section
- LA = Lateral section
- AL = Anterolateral section
- A = Anterior section

- $\sum$ = Total amount of rib fractures in the specific subgroup

Overall collective (n = 380, 9.8 fpp)

R

Σ	P	PL	LA	AL	A	RL
64	19		36		9	**1**
120	1	34	26	56	3	**2**
187	7	50	65	61	4	**3**
212	16	49	71	73	3	**4**
236	17	52	84	82	1	**5**
229	30	47	83	68	1	**6**
194	29	44	81	40	0	**7**
167	35	48	76	8	0	**8**
138	41	54	43	0	0	**9**
96	31	47	18	0	0	**10**
50	22	24	4	0	0	**11**
22	11	11	0	0	0	**12**
Σ **1715**	259	460	587	388	21	

L

RL	A	AL	LA	PL	P	Σ
**1**	10		35		31	76
**2**	1	62	32	42		137
**3**	3	73	68	55	7	206
**4**	2	82	93	68	17	262
**5**	2	91	91	77	19	280
**6**	0	78	98	65	36	277
**7**	0	46	94	74	41	255
**8**	0	15	73	75	37	200
**9**	0	1	44	71	39	155
**10**	0	0	16	44	29	89
**11**	0	0	2	32	23	57
**12**	0	0	0	13	13	26
	18	448	646	616	292	**2020**

> 50%

Cardiopulmonary resuscitation (n = 33, 9.1 fpp)

R | L

Σ	P	PL	LA	AL	A	RL	A	AL	LA	PL	P	Σ
3	1		1		1	**1**	0		2		4	6
17	0	1	0	15	1	**2**	0	18	2	0	0	20
24	0	0	6	17	1	**3**	2	20	5	1	0	28
26	0	1	3	21	1	**4**	1	21	9	1	0	32
25	0	0	3	22	0	**5**	1	23	6	1	0	31
19	0	0	1	18	0	**6**	0	17	6	3	1	27
11	0	0	2	9	0	**7**	0	7	5	1	2	15
2	0	0	1	1	0	**8**	0	3	4	1	0	7
1	0	0	1	0	0	**9**	0	0	0	1	0	1
2	1	0	1	0	0	**10**	0	0	1	0	0	1
1	1	0	0	0	0	**11**	0	0	0	0	0	0
1	1	0	0	0	0	**12**	0	0	0	0	0	0
Σ **132**	4	2	19	103	4		4	109	40	9	7	**169**

> 50%

Car/truck accident (n = 75, 8.9 fpp)

R | L

∑	P	PL	LA	AL	A	RL	A	AL	LA	PL	P	∑
17	3		12		2	**1**	1		12		4	17
17	0	2	8	6	1	**2**	0	12	4	2	0	18
32	0	4	16	12	0	**3**	1	17	8	5	0	31
41	0	6	23	12	0	**4**	1	21	12	2	1	37
47	2	4	27	14	0	**5**	1	18	17	7	1	44
47	6	3	23	15	0	**6**	0	17	21	7	2	47
40	4	2	23	11	0	**7**	0	12	23	9	4	48
36	6	2	26	2	0	**8**	0	3	21	9	3	36
24	7	4	13	0	0	**9**	0	1	15	10	5	31
13	3	5	5	0	0	**10**	0	0	5	7	4	16
6	2	3	1	0	0	**11**	0	0	0	8	4	12
2	0	2	0	0	0	**12**	0	0	0	3	3	6
∑ **322**	33	37	177	72	3		4	101	138	69	31	**343**

> 50%

Motorcycle acc. (collisions/falls) (n = 42, 9.9 fpp)

R | L

Σ	P	PL	LA	AL	A	RL	A	AL	LA	PL	P	Σ
9	2		5		2	**1**	3		4		3	10
15	1	5	2	6	1	**2**	0	4	3	7	0	14
22	2	10	4	4	2	**3**	0	4	8	9	1	22
20	3	6	6	4	1	**4**	0	6	13	12	5	36
19	2	9	4	4	0	**5**	0	6	11	13	5	35
21	2	10	3	6	0	**6**	0	6	14	8	9	37
15	2	7	4	2	0	**7**	0	3	12	11	11	37
15	4	6	3	2	0	**8**	0	2	7	11	7	27
14	4	8	2	0	0	**9**	0	0	4	6	11	21
7	2	4	1	0	0	**10**	0	0	1	4	5	10
2	1	1	0	0	0	**11**	0	0	0	3	2	5
1	1	0	0	0	0	**12**	0	0	0	0	1	1
Σ **160**	26	66	34	28	6		3	31	77	84	60	**255**

> 50%

Bicycle accident (n = 14, 10.7 fpp)

Σ	P	PL	LA	AL	A	RL	A	AL	LA	PL	P	Σ
R												L
2	0		1		1	**1**	1		3		3	7
6	0	3	1	2	0	**2**	0	1	1	4	0	6
8	0	3	5	0	0	**3**	0	3	2	5	0	10
8	1	1	6	0	0	**4**	0	2	5	5	1	13
8	1	3	3	1	0	**5**	0	1	5	5	0	11
8	1	3	4	0	0	**6**	0	0	3	6	1	10
8	1	4	2	1	0	**7**	0	1	4	4	3	12
5	0	4	1	0	0	**8**	0	0	2	5	3	10
5	0	4	1	0	0	**9**	0	0	1	2	2	5
2	0	2	0	0	0	**10**	0	0	0	2	2	4
1	1	0	0	0	0	**11**	0	0	0	1	0	1
0	0	0	0	0	0	**12**	0	0	0	0	0	0
Σ **61**	5	27	24	4	1		1	8	26	39	15	**89**

> 50%

R Pedestrian collisions with vehicle (n = 13, 12.8 fpp) L

Σ	P	PL	LA	AL	A	RL	A	AL	LA	PL	P	Σ
	1		3		1	**1**	0		0		1	
7	0	1	2	4	0	**2**	0	1	2	2	0	5
9	0	2	5	2	0	**3**	0	2	5	4	0	11
10	1	1	5	3	0	**4**	0	2	6	5	0	13
11	0	2	9	0	0	**5**	0	0	8	5	0	13
10	1	2	7	0	0	**6**	0	2	6	4	1	13
5	0	1	4	0	0	**7**	0	1	5	6	1	13
4	0	1	3	0	0	**8**	0	0	5	6	0	11
3	0	3	0	0	0	**9**	0	0	3	5	0	8
2	0	1	1	0	0	**10**	0	0	2	5	0	7
0	0	0	0	0	0	**11**	0	0	0	2	2	4
1	1	0	0	0	0	**12**	0	0	0	0	1	1
Σ **67**	4	14	39	9	1		0	8	42	44	6	**100**

> 50%

Fall from bicycle (n = 21, 9.0 fpp)

R | L

Σ	P	PL	LA	AL	A	RL	A	AL	LA	PL	P	Σ
1	1		0		0	**1**	0		3		2	5
7	0	2	3	2	0	**2**	0	2	1	6	0	9
13	0	6	5	2	0	**3**	0	1	3	5	0	9
14	3	4	2	5	0	**4**	0	3	3	6	0	12
13	1	5	5	2	0	**5**	0	7	2	8	0	17
18	4	4	7	3	0	**6**	0	3	3	6	1	13
12	4	1	6	1	0	**7**	0	2	2	5	1	10
8	5	2	1	0	0	**8**	0	0	2	3	1	6
6	3	0	3	0	0	**9**	0	0	1	4	0	5
3	3	0	0	0	0	**10**	0	0	1	3	0	4
0	0	0	0	0	0	**11**	0	0	0	2	1	3
0	0	0	0	0	0	**12**	0	0	0	1	0	1
Σ **95**	24	24	32	15	0		0	18	21	49	6	**94**

> 50%

Fall from low height (< 3 m) (n = 96, 9.7 fpp)

R | L

Σ	P	PL	LA	AL	A	RL	A	AL	LA	PL	P	Σ
9	2		6	1		**1**	1		4	7		12
23	0	10	4	9	0	**2**	1	9	5	10	0	25
36	1	14	10	11	0	**3**	0	11	20	13	1	45
46	5	15	15	11	0	**4**	0	12	23	17	3	55
63	6	16	22	18	1	**5**	0	11	20	20	6	57
57	9	13	26	8	1	**6**	0	13	26	15	10	64
59	12	18	23	6	0	**7**	0	6	21	20	8	55
55	13	19	22	1	0	**8**	0	3	15	22	11	51
53	19	19	15	0	0	**9**	0	0	9	22	12	43
41	16	18	7	0	0	**10**	0	0	2	12	8	22
24	14	8	2	0	0	**11**	0	0	0	10	4	14
10	5	5	0	0	0	**12**	0	0	0	5	4	9
Σ **476**	102	155	152	64	3		2	65	145	166	74	**452**

> 50%

Fall from great height (≥ 3 m) (n = 45, 10.8 fpp)

R | L

Σ	P	PL	LA	AL	A	RL	A	AL	LA	PL	P	Σ
7	4		3		0	**1**	3		3	4		10
16	0	6	3	7	0	**2**	0	8	7	6	0	21
24	2	9	5	7	1	**3**	0	6	9	8	2	25
24	2	9	4	8	1	**4**	0	6	11	13	5	35
23	3	9	3	8	0	**5**	0	10	12	13	5	40
21	3	8	3	7	0	**6**	0	9	13	13	6	41
20	2	9	5	4	0	**7**	0	3	13	13	8	37
22	6	8	6	2	0	**8**	0	0	10	13	10	33
15	6	8	1	0	0	**9**	0	0	6	13	6	25
12	5	7	0	0	0	**10**	0	0	1	5	7	13
10	3	7	0	0	0	**11**	0	0	0	2	6	8
3	2	1	0	0	0	**12**	0	0	0	1	2	3
Σ **197**	38	81	33	43	2		3	42	85	100	61	**291**

> 50%

Squashing/burying accident (n = 13, 15.8 fpp)

R

Σ	P	PL	LA	AL	A	RL
7	2		4	1		**1**
8	0	2	2	4	0	**2**
10	1	0	6	3	0	**3**
12	0	3	4	5	0	**4**
15	1	2	5	7	0	**5**
17	2	2	6	7	0	**6**
12	2	0	6	4	0	**7**
10	1	4	5	0	0	**8**
6	1	4	1	0	0	**9**
4	1	2	1	0	0	**10**
2	0	2	0	0	0	**11**
2	0	2	0	0	0	**12**
Σ **105**	11	23	40	30	1	

L

RL	A	AL	LA	PL	P	Σ
**1**	1		2	1		4
**2**	0	3	2	3	0	8
**3**	0	5	3	2	0	10
**4**	0	4	5	3	0	12
**5**	0	7	3	2	0	12
**6**	0	4	3	1	2	10
**7**	0	3	6	3	1	13
**8**	0	2	4	3	1	10
**9**	0	0	2	6	1	9
**10**	0	0	0	4	1	5
**11**	0	0	1	2	2	5
**12**	0	0	0	3	0	3
Σ	1	28	31	32	9	**101**

> 50%

Patients with Flail Chest (n = 44, 12.6 fpp)

R: Σ	P	PL	LA	AL	A	RL	A	AL	LA	PL	P	Σ :L
10	1		8		1	**1**	0		8	1		9
16	1	6	4	4	1	**2**	1	10	5	7	0	23
26	2	8	10	4	2	**3**	2	10	13	8	1	34
30	4	9	8	7	2	**4**	1	8	18	14	2	43
34	6	5	14	9	0	**5**	1	12	13	12	2	40
32	8	7	11	6	0	**6**	0	8	18	8	5	39
30	7	4	14	5	0	**7**	0	7	11	12	6	36
25	7	8	8	2	0	**8**	0	2	8	14	7	31
21	7	7	7	0	0	**9**	0	0	6	11	8	25
13	5	4	4	0	0	**10**	0	0	0	8	6	14
7	4	2	1	0	0	**11**	0	0	0	4	4	8
3	1	2	0	0	0	**12**	0	0	0	3	2	5
Σ **247**	53	62	89	37	6		5	57	100	101	44	**307**

> 50%

Patients w/o Flail Chest (n = 336, 9.5 fpp)

R

∑	P	PL	LA	AL	A	RL
54	18		28		8	**1**
104	0	28	22	52	2	**2**
161	5	42	55	57	2	**3**
182	12	40	63	66	1	**4**
202	11	47	70	73	1	**5**
197	22	40	72	62	1	**6**
164	22	40	67	35	0	**7**
142	28	40	68	6	0	**8**
117	34	47	36	0	0	**9**
83	26	43	14	0	0	**10**
43	18	22	3	0	0	**11**
19	10	9	0	0	0	**12**
∑ **1468**	206	398	498	351	15	

L

RL	A	AL	LA	PL	P	∑
**1**	10		27	30		67
**2**	0	52	27	35	0	114
**3**	1	63	55	47	6	172
**4**	1	74	75	54	15	219
**5**	1	79	78	65	17	240
**6**	0	70	80	57	31	238
**7**	0	39	83	62	35	219
**8**	0	13	65	61	30	169
**9**	0	1	38	60	31	130
**10**	0	0	16	36	23	75
**11**	0	0	2	28	19	49
**12**	0	0	0	10	11	21
	13	391	546	515	248	**1713**

> 50%

Transverse fractures (n = 662)

R | L

Σ	P	PL	LA	AL	A	RL	A	AL	LA	PL	P	Σ
22	4		13	5		**1**	6		13	6		25
17	0	5	5	5	2	**2**	0	12	5	14	0	31
36	0	10	14	10	2	**3**	1	11	16	15	1	44
38	1	12	11	12	2	**4**	1	14	23	12	3	53
40	1	10	23	6	0	**5**	1	18	21	18	3	61
44	7	12	17	8	0	**6**	0	7	15	16	4	42
28	6	12	9	1	0	**7**	0	3	13	10	6	32
24	7	12	5	0	0	**8**	0	0	9	15	7	31
23	6	11	6	0	0	**9**	0	0	4	14	5	23
10	2	6	2	0	0	**10**	0	0	1	5	4	10
7	2	3	2	0	0	**11**	0	0	0	7	8	15
2	1	1	0	0	0	**12**	0	0	0	1	3	4
Σ **291**	37	94	107	42	11		9	65	120	127	50	**371**

> 50%

Oblique fractures (n = 940)

R Σ	P	PL	LA	AL	A	RL	A	AL	LA	PL	P	L Σ
22	8		13		1	**1**	2		13		10	25
30	0	9	7	13	1	**2**	1	12	10	9	0	32
41	2	13	14	11	1	**3**	0	13	11	14	1	39
41	4	9	18	10	0	**4**	0	8	26	18	5	57
60	7	17	20	16	0	**5**	0	9	24	23	6	62
58	8	14	25	11	0	**6**	0	13	32	19	5	69
57	6	18	30	3	0	**7**	0	4	31	26	5	66
47	5	16	25	1	0	**8**	0	2	19	26	8	55
38	13	12	13	0	0	**9**	0	0	10	28	9	47
28	9	13	6	0	0	**10**	0	0	7	19	4	30
10	4	4	2	0	0	**11**	0	0	1	11	3	15
4	2	2	0	0	0	**12**	0	0	0	5	2	7
Σ **436**	68	127	173	65	3		3	61	184	198	58	**504**

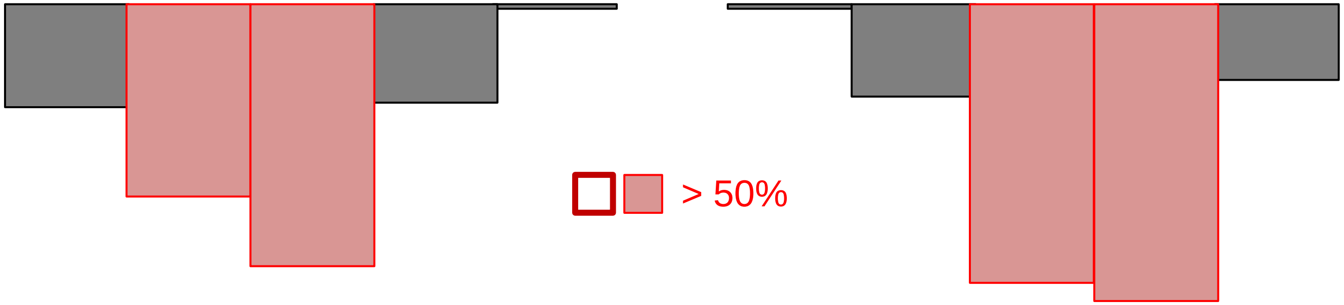

> 50%

Multifragment fractures (n = 356)

R

∑	P	PL	LA	AL	A	RL
8	3		5		0	**1**
12	0	4	3	5	0	**2**
16	1	7	6	2	0	**3**
19	2	7	8	2	0	**4**
19	1	5	11	2	0	**5**
17	4	4	7	2	0	**6**
19	4	6	8	1	0	**7**
8	4	3	1	0	0	**8**
21	8	11	2	0	0	**9**
10	3	6	1	0	0	**10**
6	3	3	0	0	0	**11**
2	1	1	0	0	0	**12**
∑ **157**	34	57	52	14	0	

L

RL	A	AL	LA	PL	P	∑
**1**	0		7	4		11
**2**	0	3	2	4	0	9
**3**	1	1	9	9	0	20
**4**	0	3	7	11	3	24
**5**	0	3	9	15	1	28
**6**	0	5	7	10	7	29
**7**	0	2	7	11	8	28
**8**	0	0	3	5	5	13
**9**	0	0	1	5	7	13
**10**	0	0	0	4	8	12
**11**	0	0	0	2	5	7
**12**	0	0	0	2	2	4
	1	17	52	78	50	**198**

> 50%

Infractions (n = 1632)

R Σ	P	PL	LA	AL	A	RL	A	AL	LA	PL	P	L Σ
12	4		5		3	**1**	2		2		11	15
58	1	15	11	31	0	**2**	0	33	13	15	0	61
87	4	19	26	37	1	**3**	0	45	28	17	4	94
107	9	19	32	46	1	**4**	0	57	33	25	5	120
107	8	18	28	52	1	**5**	0	57	33	21	7	118
101	11	16	32	41	1	**6**	0	51	41	19	17	128
80	13	7	31	30	0	**7**	0	34	43	26	20	123
78	17	16	40	5	0	**8**	0	13	40	29	17	98
44	12	16	16	0	0	**9**	0	1	27	21	17	66
38	14	18	6	0	0	**10**	0	0	6	14	12	32
24	12	12	0	0	0	**11**	0	0	1	9	8	18
14	7	7	0	0	0	**12**	0	0	0	5	4	9
Σ **750**	112	163	227	241	7		2	291	267	201	121	**882**

> 50%

No Dislocation (n = 2015)

R | L

Σ	P	PL	LA	AL	A	RL	A	AL	LA	PL	P	Σ
28	12		12		4	**1**	4		10		18	32
67	1	19	14	33	0	**2**	0	36	18	22	0	76
99	4	22	33	39	1	**3**	1	49	35	22	6	113
121	12	24	35	49	1	**4**	1	59	40	28	12	140
131	11	24	36	59	1	**5**	1	62	42	27	11	143
121	15	20	37	48	1	**6**	0	55	49	26	21	151
107	16	17	38	36	0	**7**	0	37	45	30	29	141
99	23	20	49	7	0	**8**	0	13	44	34	22	113
68	17	26	25	0	0	**9**	0	1	30	32	23	86
56	21	24	11	0	0	**10**	0	0	8	20	14	42
28	14	14	0	0	0	**11**	0	0	1	12	9	22
15	8	7	0	0	0	**12**	0	0	0	5	6	11
Σ **945**	154	217	290	271	8		7	312	322	258	171	**1070**

> 50%

Dislocation < rib width (n = 1174)

R Σ	R P	R PL	R LA	R AL	R A	RL	L A	L AL	L LA	L PL	L P	L Σ
23	7		14		2	**1**	5		21		12	38
38	0	10	9	17	2	**2**	1	16	10	12	0	39
54	2	20	14	15	3	**3**	2	10	24	22	1	59
55	2	17	20	15	1	**4**	1	12	40	24	4	81
72	5	18	33	16	0	**5**	1	20	33	38	7	99
83	14	19	33	17	0	**6**	0	20	31	26	12	89
63	11	20	28	4	0	**7**	0	9	36	26	9	80
42	7	19	15	1	0	**8**	0	2	16	23	11	52
54	16	22	16	0	0	**9**	0	0	11	24	12	47
25	5	15	5	0	0	**10**	0	0	5	15	12	32
15	7	4	4	0	0	**11**	0	0	1	10	10	21
4	2	2	0	0	0	**12**	0	0	0	6	2	8
Σ **528**	78	166	191	85	8		10	89	228	226	92	**645**

> 50%

Dislocation > rib width (n = 546)

	R: Σ	P	PL	LA	AL	A	RL	A	AL	LA	PL	P	L: Σ
	13	0		10	3		**1**	1		4	1		6
	14	0	4	3	6	1	**2**	0	10	4	8	0	22
	33	1	7	18	7	0	**3**	0	14	9	11	0	34
	35	2	7	16	9	1	**4**	0	11	13	16	1	41
	32	1	9	15	7	0	**5**	0	9	16	12	1	38
	24	1	7	13	3	0	**6**	0	3	18	13	3	37
	23	2	6	15	0	0	**7**	0	0	13	17	3	33
	26	5	9	12	0	0	**8**	0	0	13	18	4	35
	16	8	6	2	0	0	**9**	0	0	3	15	4	22
	15	5	8	2	0	0	**10**	0	0	3	9	3	15
	7	1	6	0	0	0	**11**	0	0	0	10	4	14
	3	1	2	0	0	0	**12**	0	0	0	2	5	7
Σ	**241**	27	71	106	32	5		1	47	96	131	29	**304**

> 50%

R

RFAW vertebral body fractures (n = 170)

L

Σ	P	PL	LA	AL	A	RL	A	AL	LA	PL	P	Σ
2	2		0		0	**1**	0		0		1	1
3	0	2		1	0	**2**	0	3	2	1	0	6
5	0	0	2	2	1	**3**	0	2	2	4	0	8
8	2	2	3	1	0	**4**	0	3	2	6	1	12
5	0	3	0	2	0	**5**	0	3	2	6	1	12
6	1	1	3	1	0	**6**	0	2	6	7	1	16
10	2	2	6	0	0	**7**	0	1	6	5	4	16
13	4	4	4	1	0	**8**	0	1	4	6	3	14
6	4	1	1	0	0	**9**	0	0	3	2	4	9
2	2	0	0	0	0	**10**	0	0	0	2	2	4
5	2	3	0	0	0	**11**	0	0	0	2	3	5
1	0	1	0	0	0	**12**	0	0	0	0	1	1
Σ **66**	19	19	19	8	1		0	15	27	41	21	**104**

> 50%

R

RFAW transverse process fractures (n = 103)

L

∑	P	PL	LA	AL	A	RL	A	AL	LA	PL	P	∑
6	1		5		0	**1**	1		0	3		4
1	0	1	0	0	0	**2**	0	0	2	0	0	2
1	0	1	0	0	0	**3**	0	0	2	2	0	4
4	2	1	1	0	0	**4**	0	0	1	2	0	3
9	4	1	3	1	0	**5**	0	1	0	4	1	6
9	3	2	3	1	0	**6**	0	2	2	1	3	8
3	1	0	1	1	0	**7**	0	1	1	4	3	9
4	2	0	2	0	0	**8**	0	0	0	4	3	7
2	0	0	2	0	0	**9**	0	0	0	4	3	7
3	1	1	1	0	0	**10**	0	0	0	4	2	6
1	0	0	1	0	0	**11**	0	0	0	1	1	2
1	0	1	0	0	0	**12**	0	0	0	1	0	1
∑ **44**	14	8	19	3	0		1	4	8	27	19	**59**

> 50%

RFAW sternal fractures (n = 469)

R | L

Σ	P	PL	LA	AL	A	RL	A	AL	LA	PL	P	Σ
16	5		8		3	**1**	3		12		8	23
26	0	8	4	13	1	**2**	0	18	9	2	0	29
37	0	7	14	13	3	**3**	2	19	9	5	1	36
38	2	8	11	14	3	**4**	1	17	12	6	1	37
43	2	7	18	15	1	**5**	1	19	15	7	2	44
41	4	7	17	12	1	**6**	0	15	15	5	3	38
28	2	5	12	9	0	**7**	0	7	15	5	6	33
0	0	0	0	0	0	**8**	0	0	0	0	0	0
0	0	0	0	0	0	**9**	0	0	0	0	0	0
0	0	0	0	0	0	**10**	0	0	0	0	0	0
0	0	0	0	0	0	**11**	0	0	0	0	0	0
0	0	0	0	0	0	**12**	0	0	0	0	0	0
Σ **229**	15	42	84	76	12		7	95	87	30	21	**240**

> 50%

RFAW clavicle fractures (n = 8)

R | L

	∑	P	PL	LA	AL	A	RL	A	AL	LA	PL	P	∑
	6	0		5		1	**1**	0		1		1	2
	0	0	0	0	0	0	**2**	0	0	0	0	0	0
	0	0	0	0	0	0	**3**	0	0	0	0	0	0
	0	0	0	0	0	0	**4**	0	0	0	0	0	0
	0	0	0	0	0	0	**5**	0	0	0	0	0	0
	0	0	0	0	0	0	**6**	0	0	0	0	0	0
	0	0	0	0	0	0	**7**	0	0	0	0	0	0
	0	0	0	0	0	0	**8**	0	0	0	0	0	0
	0	0	0	0	0	0	**9**	0	0	0	0	0	0
	0	0	0	0	0	0	**10**	0	0	0	0	0	0
	0	0	0	0	0	0	**11**	0	0	0	0	0	0
	0	0	0	0	0	0	**12**	0	0	0	0	0	0
∑	**6**	0	0	5	0	1		0	0	1	0	1	**2**

> 50%
